# Health professionals’ knowledge on dengue and health facility preparedness for case detection: A cross-sectional study in Dar es Salaam, Tanzania

**DOI:** 10.1371/journal.pntd.0011761

**Published:** 2023-11-21

**Authors:** Ummul-khair Mustafa, Elingarami Sauli, Johanna Brinkel, Katharina Sophia Kreppel

**Affiliations:** 1 School of Life Science and Bioengineering, Nelson Mandela African Institution of Science and Technology, Arusha, Tanzania; 2 Department of Infectious Disease Epidemiology, Bernhard Nocht lnstitute for Tropical Medicine, Hamburg, Germany; 3 Department of Public Health, Institute of Tropical Medicine, Antwerpen, Belgium; Australian Red Cross Lifelood, AUSTRALIA

## Abstract

Dengue presents a growing public health concern in the Dar es Salaam region of Tanzania, marked by the recurring incidence of outbreaks. Unfortunately, there is little information available on the region’s preparedness in terms of health care workers’ knowledge on dengue as well as the availability of reagents and equipment essential for diagnosing and monitoring of dengue infections. To elucidate this, 78 health facilities were visited in Temeke district and structured questionnaires were distributed to 324 health care workers. The aim was to evaluate health care workers’ knowledge on dengue and to assess the availability of reagents and equipment essential for diagnosing and monitoring of dengue infections. Content validity of the questionnaire was achieved through extensive literature review and it exhibited high reliability (Cronbach Alpha coefficient = 0.813). Cumulative scores for responses on knowledge questions by health care workers were computed. Characteristics such as level of education, place of work and gender were tested for association with these scores using chi-square tests and logistics regression. Almost all health care workers (99.7%) were aware of dengue disease. However, less than half (46.9%) had knowledge scores of or over 40%. Clinicians had approximately four times higher knowledge scores than other cadres (AOR, 3.637; p-value≤ 0.0001), and those who worked in private facilities had twice the knowledge score than those working in government institutions (AOR, 2.071; p-value = 0.007). Only 8.6%, 35.6% and 14.7% of respondents reported the availability of dengue rapid tests, medical guidelines and refresher training respectively, showing a lack of health facilities readiness for the detection of dengue infections. Based on findings from this study, we recommend government authorities to build capacity of health care workers, to improve their understanding of dengue. We also urge the government and stakeholders to work together to ensure availability of diagnostic tests and other tools needed for diagnosis and surveillance of dengue.

## Author summary

Dengue is a growing public health concern in Tanzania, particularly in the Dar es Salaam region, where outbreaks are common. However, little is known about the region’s preparedness for dengue outbreaks, including the knowledge of health care workers and availability of diagnostic equipment. In a study of 324 health care workers in 78 health facilities in Temeke district, it was found that while almost all workers were aware of dengue, less than half had adequate knowledge of diagnosis, management, and monitoring. Clinicians had higher knowledge scores than other cadres, and those working in private facilities had more knowledge than those in government institutions. Only a small percentage of respondents reported the availability of diagnostic tests, medical guidelines, and refresher training. The study recommends building the capacity of health care workers and ensuring the availability of diagnostic tests and tools to improve readiness for dengue outbreaks. Dengue outbreaks occur every two years in Tanzania, with the last outbreak in 2019 resulting in over 6,800 cases and 13 deaths. As climate change is expected to increase the number of dengue cases, improving health care workers’ knowledge and diagnostic capacity is crucial to effectively detect outbreaks.

## Introduction

Dengue is a mosquito-borne disease that has become a major public health concern globally [1]. It is transmitted by the *Aedes* mosquito which is expanding its distribution. The number of dengue cases has increased dramatically in the past two decades, with a rise from 505,430 to 5.2 million cases reported between 2000 and 2019 [2]. Dengue is caused by the dengue virus (DENV), a member of the Flaviviridae family [2]. The disease affects up to 400 million people per year and is endemic in 128 countries, mostly in the Americas and Asia, but also in 34 countries in Africa [2, 3].

While first infection in humans is usually mild, a second infection with another dengue virus strain can lead to serious morbidity and mortality, causing economic loss to individual patients and their families, as well as to the health system [4–6]. Dengue illness can occur as either a mild infection, dengue fever, or severe disease, such as dengue hemorrhagic fever and hypovolemic shock, which can be fatal [7, 8].

Currently, a vaccine for dengue is only available in some countries for people that have already been infected previously and can afford the cost [2], and there is still no specific treatment for dengue [7, 9]. Control measures include eradicating *Aedes* breeding sites and implementing bite prevention methods [2]. To focus interventions, a strong dengue surveillance system is required in endemic countries for monitoring the burden of dengue and planning effective control actions [10].

In Tanzania, dengue outbreaks occur repeatedly at least every two years, so far affecting mostly the coastal region of Dar es salaam [3, 11]. During the 2019 outbreak, 6,873 cases of dengue were officially reported with 13 deaths [11], but it is likely that the actual number of cases and deaths were higher due to the limited capacity for proper surveillance, preparedness, and access to diagnostic tests [12, 13]. When patients are reporting to their health care centers with febrile illness symptoms, malaria remains the most likely diagnosis given in the absence of confirmatory tests [14, 15]. According to the Tanzania infectious disease surveillance and response system, any patient with symptoms such as mild/severe fever, bleeding from nose, gums, vagina, skin or eyes, vomiting blood, and impaired consciousness meets the criteria for suspected dengue and should be reported to district health officers within 24 hours for prompt response [16]. While there is no direct treatment for dengue, vector control measures are used to control transmission after an outbreak is reported. However, despite the existing guidelines for disease surveillance and response, the system is not well implemented, leading to underreporting and missed cases of dengue [12, 17, 18], resulting in a lack of transmission control measures being carried out.

Due to the effect of climate change in Tanzania [19], dengue cases in the Dar es salaam region are expected to rise in the future and are predicted to spread across the country [20, 21]. To prepare the country for this, there is an urgent need to understand challenges and identify weaknesses in the health care service delivery chain to be able to improve the situation. Health care workers are at the front line of the war against dengue and other emerging and re-emerging infectious diseases; hence an understanding of their current knowledge of dengue is essential if the detection and reporting of the disease is to be improved. Previous research findings from the northern Kilimanjaro region, Tanzania, reported insufficient knowledge by health care providers on the management of dengue [22, 23]. Limited information also exists even from the hotspot regions like Dar es Salaam [12]. The aim of this study was, therefore, to assess health care workers’ knowledge about cause, transmission, diagnosis, prevention, management and surveillance of dengue in Dar es Salaam. The study also included assessment of health facilities preparedness for dengue in terms of availability of reagents and equipments needed for detection of dengue cases. The findings from this study will assist in the planning and implementation of dengue intervention programmes and surveillance by health professionals in the Dar es Salaam region and elsewhere in Tanzania.

## Methods

### Ethical approval

The study was approved by the Tanzania Northern Zone Health Research Ethics Committee (KNCHREC). Research Proposal No: KNCHREC00061/12/2021, issued: 31st January 2022.

### Study area

**Fig 1:**
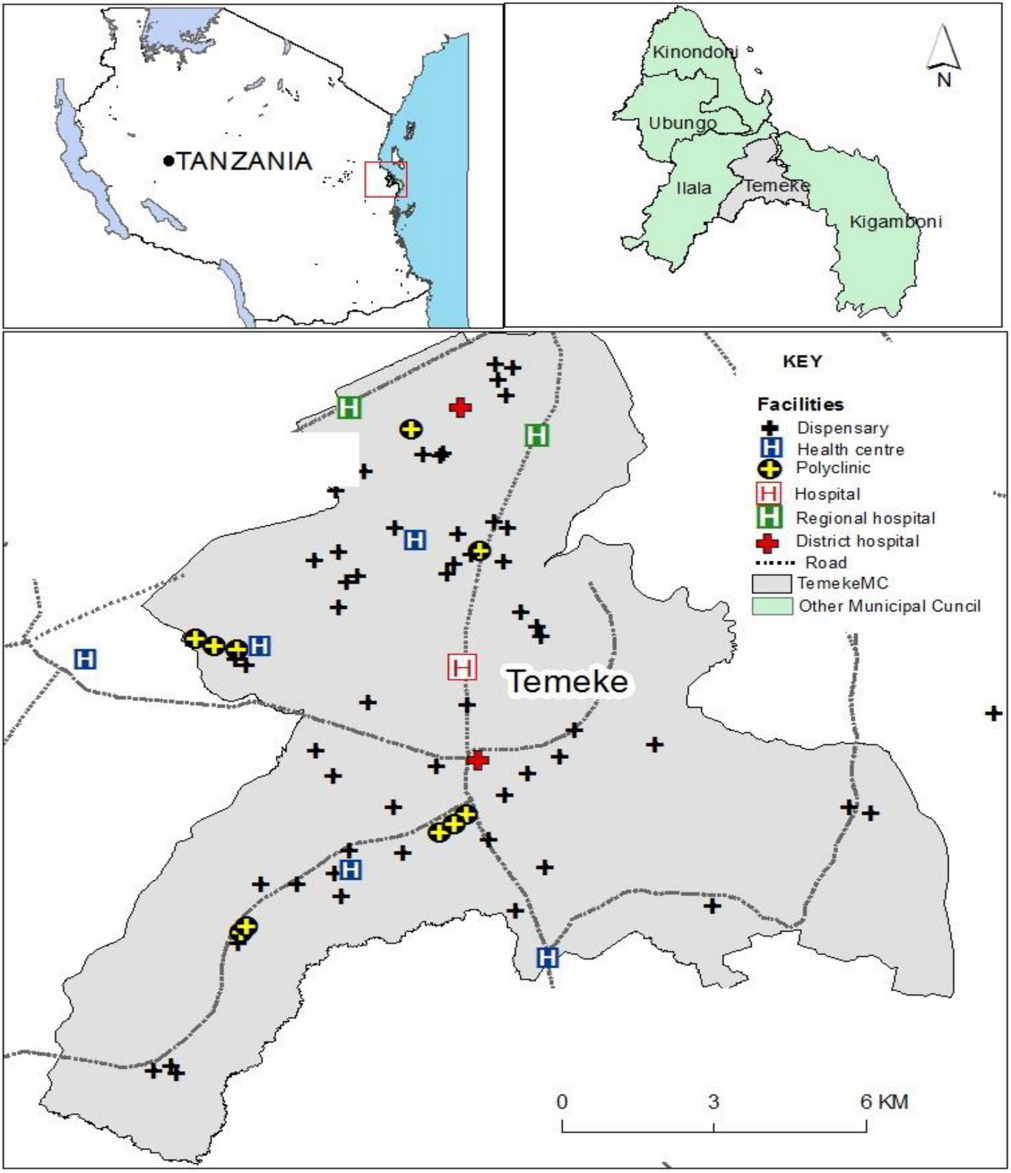
Map displaying the surveyed health facilities in Temeke district, Dar es Salaam region, Tanzania. This map was generated using ArcGIS version 11.1, license number EFL96303636612. The map base layers were sourced from the National Bureau of Statistics (https://www.nbs.go.tz/index.php/en/census-surveys/gis/568-tanzania-districtsshapefiles-2019). Licence information is available at (https://www.nbs.go.tz/nbs/takwimu/references/Licence-Agreement-NBS.pdf).

The study was conducted in Dar es Salaam region, Tanzania. Dar es Salaam region experiences regular dengue outbreaks [24]. Specifically, the research was conducted within the Temeke district of Dar es Salaam (Fig 1). Temeke district was selected randomly from a list of five administrative districts namely [12]: Ilala, Kigamboni, Kinondoni, Temeke and Ubungo. Dar es Salaam region is found at 6°51′S, 39°18′E along the south-western coast of the Indian Ocean [25]. The region has an area of 1,339 km^2^, the climate is generally hot and humid, with a mean daily temperature of 26°C, up to 1100 mm annual rainfall, and up to 100% relative humidity at night and bimodal rainfall patterns with a long rainy season from March to May and short rains from October to December [26]. The climatic conditions are favourable for dengue transmission and from the year 2010 to date, the Dar es Salaam region reported five dengue outbreaks (2010, 2013, 2014, 2018, 2019) [24].

### Study population and study design

This study aimed to investigate health care workers’ knowledge and facility preparedness in diagnosing, managing, and surveilling dengue fever in Temeke district, Dar es Salaam region, Tanzania. The study was a health facility-based cross-sectional survey that took place between November and December 2022.

In this study, the study population consisted of clinicians, laboratory personnel, nurses and medical attendants. Among health cadres, clinicians were chosen because they are frontline in diagnosis and management of illnesses including dengue. Health cadres such as nurses, laboratory personnel and medical attendants were included in the study due to the reason that they provide supportive functions that significantly influence patient outcomes and diagnostics [27]. In health-staff-short countries like Tanzania, task shifting is common, where laboratory personnel’s, medical attendants and nurses often take on clinician roles [28–31].

The approach used to reach health care workers was to visit health facilities involved with generalized clinical services including diagnostic and management of febrile patients. Generally, the Tanzania health system is organized in a pyramid structure. Dispensaries represent the first contact and offer outpatient basic services [32]. Health centers are the next level, provide outpatient and inpatient services, and receive referrals from dispensaries. Hospitals are at the top of the hierarchy; provide outpatient, inpatient and specialized services and receive referrals from health centers [32]. In addition, there are facilities registered as clinics and provide either specialized or general clinical services and send referral cases to health centers or hospitals [33]. Another category of health facilities are medical diagnostic laboratories which mainly offer laboratory investigations [33].

As with other illnesses, patients with suspected dengue typically visit dispensaries or general clinics first and serious cases are referred by clinicians to health centers and hospitals. However, bypassing of primary health facilities and self-referral to hospitals are common practice [34–36]. Taking this into consideration, we recruited health workers across different types of health facilities as they have equal chance encountering suspect dengue cases at the outpatient department.

### Sample size determination and sampling

The sample size of health care workers was obtained by using the formula for cross-sectional surveys (n = z^2^ P (100-P)/ ε^2^) [37]. Standard normal deviate (z) of 1.96 with 95% confidence interval, knowledge prevalence (p) of 74.1% as reported from a previous study in the Kilimanjaro region, Tanzania [22], Margin of error (ε) of 0.05 and an addition of 10% non-response rate were used to compute the sample size of 324 health care workers.

A multi-stage sampling process was followed: First, one district (Temeke) was randomly selected from five districts of Dar es Salaam region. A list of 166 health care facilities present in the Temeke district was obtained from the district medical officer. They consisted of 24 public health facilities and 142 private health facilities.

Health facilities were screened for eligibility criteria and 149 facilities were eligible to participate in the study. Among these, 22 were public health facilities while the remaining 127 were private health facilities. Inclusion criteria were being registered to provide generalized clinical services including diagnosis and management of febrile conditions. Exclusion criteria were being a health facility specialized in eye care, dental care, diabetes management, and laboratory diagnostics without treatment. The second exclusion criteria were being involved in the pilot testing phase of our study.

The required sample size for health care workers was 324. Convenience sampling was used to achieve this. The study recruited all 22 public health facilities meeting inclusion criteria and an additional 56 private health facilities, making 78 facilities. The selected health facilities were those easily accessible and in close proximity. Within health facilities, health workers enrolled into the study were those who were available at the day of the health facility visit. The inclusion criteria were health care workers who were 18 years of age or older, willing to sign the consent form, and complete the questionnaire within 24 hours. The exclusion criteria were participants who refused to provide their written consent or failed to return the questionnaire within 24 hours of receiving it.

The choice for convenience sampling was due to limited resources in terms of both budget and man power. Convenience sampling allowed striking a balance between acquiring the data while mitigating logistical challenges. Convenient sampling is often used in health facility-based studies as it is a cost-effective, less time consuming and reliable option [38–40]. S1 Fig provides a visual representation of the sampling process.

### Questionnaire deployment and data collection

A pretested questionnaire consisting of 38 questions was used to investigate health workers’ knowledge of dengue and facility preparedness in detecting dengue cases. There was no standardized tool for assessment of knowledge for dengue among health care workers as well as facilities preparedness for dengue detection. To ensure content validity, the questionnaire development was informed by a review of World Health Organization guideline for clinical management of dengue [9], past survey of dengue knowledge among health care workers [22, 23, 37, 41–49], and other literature describing diagnostics and management of dengue [50–54]. Furthermore, a Cronbach Alpha coefficient was used to measure reliability and internal consistency (coefficient = 0.813) which indicated high reliability [55–57].

The questionnaire consisted of the following three sections. Section A for socio-demographic characteristics of respondents (10 questions) as shown in Table 1. Section B consisted of 22 questions which covered information related to knowledge on dengue. First, two questions asked for awareness of dengue and severe dengue fever. The remaining 20 questions were divided into six domains. The first domain contained one questions on clinical features of dengue fever and consisted of 11 correct responses (S1 Table). The second domain comprised 3 questions on cause, mode of transmission and preventive measures and had a total of 8 correct responses (S2 Table). The third domain consisted of 2 questions which delved into identifying warning signs and risk factors for severe dengue and comprised a total of 17 correct responses (Table 2). The fourth domain consisted of 2 questions which focused on clinical features of dengue shock syndrome and had a total of 13 correct responses (Table 3). The fifth domain consisted of 3 questions that concentrated on diagnostic approaches and clinical samples used in confirmation of dengue cases and had total of 9 correct responses (S3 Table). The last domain included 9 questions on management strategies and surveillance of dengue cases and had a total of 12 correct responses (S4 Table). In terms of percentages, the highest marks were allocated to domain 3 (24.29%) and domain 4 (18.57%). This was followed by domain 6 (17.14%) and domain 1 (15.71%), while domain 5 (12.86%) and domain 2 (11.43%) received relatively lower marks.

**Table 1:** Socio-demographic characteristics of respondents (N = 292).

Variable	Categories	Frequency (%)
**Gender**	Male	134(45.9)
	Female	158(54.1)
	Total	292(100)
**Age (years)**	< 20	3(1.0)
	20–30	174(59.6)
	31–41	68(23.3)
	42–52	31(10.6)
	53 +	16(5.5)
	Total	292(100)
**Qualification**	Medical clinician	110(37.7)
	Laboratory technician	82(28.1)
	Nurse	94(32.2)
	Medical attendant	6(2.1)
	Total	292(100)*
**Education**	University Degree	23(7.9)
	Advanced diploma	8(2.7)
	Diploma	166(56.8)
	Certificate	89(30.5)
	Short course	6(2.1)
	Total	292(100)
**Experience in health profession (years)**	<1	36(12.3)
	1–5	156(53.4)
	6–10	50(17.1)
	11+	50(17.1)
	Total	292(100)*
**Work station**	Dispensary	209(71.6)
	Health centre	29(9.9)
	Hospital	24(8.2)
	Polyclinic	30(10.3)
	Total	292(100)
**Type of facility**	Public/government	108(37.0)
	Private	184(63.0)
	Total	292(100)

*symbol means decimals have been rounded

**Table 2:** Health care workers’ knowledge on warning signs for severe dengue.

Variable	Proportion of respondent answering “Yes” (%)
**Warning signs for severe dengue**	
Enlarged liver	89(30.5)
Gums and nose bleed	111(38.0)
Blood in vomit or fecal matter	69(23.6)
Easy bruising at venipuncture site	59(20.2)
Heavy menstruation	13(4.5)
Petechiae	63(21.6)
Lethargy	143(49.0)
Restless	143(49.0)
Severe abdominal pain	73(25.0)
Persistent vomiting	123(42.1)
Ascities and pleural effusion	22(7.5)
Increased hematocrit levels	27(9.2)
Decreased platelet count	71(24.3)
Decreased white blood cell count	42(14.4)
**Risk factor for severe dengue**	
Having hypertension	48(16.4)
Having diabetes	48(16.4)
Getting a repeated dengue infection	135(46.2)

All symptoms and risk factors listed in Table 2 are correct answers

**Table 3:** Knowledge on dengue shock syndrome and associated symptoms (N = 292).

Cause and symptoms of dengue shock syndrome	Proportion of respondent answering “Yes” (%)
Hypovolemic shock occurs in dengue^+^	79(27.1)
Increased heart rate^+^	115(39.4)
Rapid breathing^+^	100(34.2)
Low blood pressure^+^	78(26.7)
Cold and wet limbs^+^	78(26.7)
Delayed capillary refill time^+^	62(21.2)
Restlessness^+^	105(36.0)
Confusion^+^	105(36.0)
Lethargy^+^	105(36.0)
Failure to make eye contact^+^	39(13.4)
Failure to respond to painful stimuli^+^	36(12.3)
Low urine output^+^	48(16.4)
Organ failure^+^	77(26.4)
I don’t know any symptom	31(10.6)

^+^ Symbol represents correct answers

Section C constituted 6 questions on health facility preparedness in detecting dengue outbreaks. Section A and section C as well as first two questions on section B were not involved in the calculation of knowledge score for respondents.

The questionnaire was prepared in English and then translated into the official national language Swahili. Despite English being the medium of instruction for medical training, Swahili language was opted for in this survey for two main reasons. English is not a mother tongue language in Tanzania and respondents might face difficulty in understanding, judging, and responding accurately in English [58,59]. In addition, enrolled participants had diverse educational backgrounds and varying English proficiency, Swahili ensures fair participation for all. Furthermore, use of a national language such as Swahili for our case demonstrates respect for the national language and culture, and consequently enhances trust and rapport with the respondents.

The questionnaire was self-administered and pretested on nine health care workers selected from three health facilities (1 dispensary, 1 health center and 1 hospital) within the Temeke district. These pilot facilities were excluded from the data collection process.

For section B, each correct answer received a score of 1 while an incorrect answer received 0. The total score for each respondent ranged between 0 and 70. For analysis, the scores were changed to 100% and thus ranged between 0 and 100percent.

### Statistical analysis

Collected data were entered and analyzed using SPSS version 23 (IBM Corp., Armonk, NY, USA). Descriptive statistics were used to summarize the data. Knowledge scores were tested for normality using Shapiro–Wilk and Kolmogorov–Smirnov tests [60]. The data were not normally distributed. Boxplots and Kruskal-Wallis tests were used to compare the knowledge score for clinicians, laboratory personnel, nurses and medical attendants across various knowledge domains. Beyond that, the study aimed to identify influence of socio-demographic variables on cumulative knowledge score of respondents. In circumstances were data are not normally distributed, the dichotomization of continuous variables such as the knowledge score for our case is a reasonable option to simplify data analysis and conduct logistic regression [61–63]. Consequently, dichotomization of the outcome variable simplifies understanding of data among different groups of audience [61]. Furthermore, dichotomized outcome variables facilitate decision making, for our case interventions to improve health care workers knowledge of dengue subject matter.

For this study, knowledge score were categorized into binary variables (score <40% and scores ≥40%). Knowledge scores <40% were regarded as poor, whereas scores ≥40% were regarded as good. Among different acceptable approaches for dichotomization of an outcome variable [61, 64], this study adopted cut-off points used by a previous study carried out in Kilimanjaro region, Tanzania [22].

Socio-economic independent variables (gender, age, education, qualification, and experience in the health profession, current work station and type of facility) were also categorized into binary variables to ease comparisons of knowledge grades within participant groups. Respondents’ gender remained male or female, age was subcategorized into <40 years and ≥40 years. Education was divided into diploma/degree versus certificate/short course, while qualification was subdivided into clinician versus laboratory personel/nurse/medical attendant, and current work station was subdivided into dispensary and health centre/hospital/polyclinic. The types of health facility were either private or public and experience variables were either ≤5 years or ≥6 years.

To compare knowledge (good vs. poor) between the various groups of health care workers, Chi-square tests were used. Univariate regression analysis was followed by multivariate analysis to assess the magnitudes of the association between the dependent variable and independent variables with significant Chi-square results [60]. In all statistical tests, a p-value ≤ 0.05 was considered significant [60].

## Results

A total of 324 health care workers were invited and gave written informed consent to participate in the knowledge survey. Of those, 292 health care workers completed the given questionnaires (90.0% response rate). Out of the 32 individuals who did not respond, 18 had incomplete questionnaires and 14 never sent them back. All of them stated that their reason for not completing the questionnaires was a shortage of time. Of the non-respondents, 10 worked at government-owned facilities, while the remaining 22 were affiliated with private health facilities. In terms of qualifications, 8 were classified as clinicians, 15 as nurses, and 9 as laboratory personnel. When categorized by work station, 23 were stationed at dispensaries, and the remaining 9 were based at polyclinics

### Socio-demographic characteristics of participants

In this study, the majority of the respondents were female (n = 158, 54.1%), medical clinicians (n = 110, 37.7%) and 20–30 years old (n = 174, 59.6%). In terms of education level, the majority of the respondents held a diploma in medicine, laboratory science or nursing (n = 166; 56.8%), and most of the participants were in the health profession for the period between 1–5 years (n = 156; 53.4%). In addition, the majority of respondents worked in private health care facilities (184; 63%) and there in dispensaries (209; 71.6%). A detailed summary of the respondent characteristics is provided in Table 1.

### Health care workers’ knowledge on dengue

#### Domain 1: Health care workers’ awareness and knowledge of symptoms of dengue fever

The study found that almost all respondents were aware of dengue (291, 99.7%). Out of the 11 correct symptoms of dengue fever provided in the list, the majority of respondents correctly selected high fever (267, 91.4%), followed by headache (223, 76.4%), joint pain (174, 59.6%), muscle pain (168, 57.4%), and loss of appetite (153, 52.4%). However, less commonly chosen symptoms were vomiting (142, 48.6%), pain behind the eyes (81, 27.7%), skin rashes (69, 23.6%), petechiae (55, 18.8%), gums and nose bleeding (53, 18.2%), and bruising at the venipuncture site (44, 15.1%) as shown in S1 Table. According to boxplots, as expected, clinicians had the highest knowledge score (Median = 63.64, Interquartile Range = 27.) followed by laboratory personnel (Median = 36.36, Interquartile Range = 36) and subsequently the nurses (Median = 36.36, Interquartile Range = 45). Medical attendants had the lowest scores (Median = 18.18, Interquartile Range = 59) as shown in S2 Fig. However, the difference in score between the groups of respondents was not statistically significant (Kruskal-Wallis test = 11.752, p-value = 0.008).

#### Domain 2: Knowledge on cause, transmission and prevention of dengue

Less than half of the respondent (128, 43.8%) correctly chose virus as the pathogen type causing dengue. The remaining respondents selected an incorrect response or chose “I don’t know” as shown in S2 Table. The majority of respondents knew that a mosquito bite transmits dengue (259, 88.7%), but very few knew about maternal transmission (30, 10.3%) and transmission via blood transfusion (46, 15.8%). Most of the respondents knew that sleeping under mosquitos’ nets and avoiding mosquito bites were correct measures to prevent dengue transmission (219, 75.0% and 175, 59.9% respectively). However, more than half of the respondents did not select covering water containers and emptying water holding containers around houses as methods for controlling dengue (166, 56.8% and 179, 61.3%). A detailed summary is provided in S2 Table.

Based on boxplots, clinicians had the highest knowledge score (Median = 50, Interquartile Range = 25), followed by laboratory personnel (Median = 50, Interquartile Range = 38) and then nurses (Median = 37.58, Interquartile Range = 25). Meanwhile medical attendants had the lowest score (Median = 37.58, Interquartile Range = 56), (S2 Fig). However, the difference in knowledge score among the various groups of respondents shown in the boxplots were not statistically significant (Kruskal-Wallis test = 3.604, p-value = 0.307)

#### Domain 3: Knowledge on warning signs and risk factors for severe dengue

As shown in Table 2, few respondents were knowledgeable on the fourteen (14) warning signs for severe dengue; lethargy (143, 49.0%), restlessness (143, 49.0%), persistent vomiting (123,42.1%), gums and nose bleed (111,38.0%), enlarged liver (89,30.5%), blood in vomit or fecal matter (69, 23.6%), easy bruising at venipuncture site (59, 20.2%), heavy menstruation (13, 4.5%), petechiae (63, 21.6%), severe abdominal pain (73, 25.0%), ascites and pleural effusion (22, 7.5%), increased hematocrit levels (27, 9.2%), decreased platelet count (71, 24.3%) and decreased white blood cell count (42, 14.4%). Only a few respondents selected hypertension (48, 16.4%) and diabetes (48, 16.4%), and second dengue infections (135, 46.2%) as risk factors for severe dengue.

Based on boxplots, clinicians had the highest knowledge score (Median = 35.29, Interquartile Range = 35) followed by laboratory personnel and nurses who had the same scores (both Median = 17.65, Interquartile Range = 24). Medical attendants had the lowest score (Median = 11.76, Interquartile Range = 26) as shown in S2 Fig. However, the difference in knowledge score shown in the boxplots was not statistically significant (Kruskal-Wallis test = 2.278, p-value = 0.517).

#### Domain 4: Knowledge on dengue shock syndrome and associated symptoms

A total of 292 participants answered questions about dengue shock and associated symptoms. However, only a small percentage of participants (27.1%, n = 79) correctly identified that hypovolemic shock can occur in dengue patients. Furthermore, the majority of respondents were not knowledgeable about several symptoms associated with dengue shock, including increased heart rate (39.4%, n = 115), restlessness (36.0%, n = 105), lethargy (36.0%, n = 105), rapid breathing (34.2%, n = 100), cold and wet limbs (26.7%, n = 78), low blood pressure (26.7%, n = 78), organ failure (26.4%, n = 77), delayed capillary refill time (21.2%, n = 62), low urine output (16.4%, n = 48), failure to make eye contact (13.4%, n = 39), and failure to respond to painful stimuli (12.3%, n = 36). Additionally, 10.6% (n = 31) of respondents chose the answer "I don’t know any symptom," as shown in Table 3.

According to boxplots, clinicians had the highest knowledge score (Median = 30.77, Interquartile Range = 46), followed by nurses (Median = 15.38, Interquartile Range = 17) and then medical attendants (Median = 11.54, Interquartile Range = 25; S2 Fig). Laboratory personnel had the lowest score (Median = 7.69 Interquartile Range = 23). However, the difference in knowledge score shown in the boxplots was not statistically significant (Kruskal-Wallis test = 1.838, p-value = 0.607).

#### Domain 5: Knowledge on the diagnostic of dengue

Rapid test was the most known procedure for diagnosing dengue (218, 74.7%). Other confirmatory tests were less known to the respondents; Enzymes Linked Immunosorbent Assay (ELISA), (46, 15.8%), Polymerase Chain Reaction (PCR) (75, 25.7%) and blood culture (50, 17.1%). In addition, the majority of respondents (216, 74.0%) chose whole blood as the sample used to diagnose dengue compared to serum (125, 42.8%) and plasma (125, 42.8%). Furthermore, most respondents (198, 67.8%) knew that a full blood picture was an additional test used in diagnosing dengue while only few (14, 4.8%) were aware of tourniquet test as shown in S3 Table.

As indicated by the boxplots, clinicians and laboratory personnel’s displayed comparable knowledge scores, both of which were the highest (Median = 44.44, Interquartile Range = 22). Nurses followed (Median = 33.33, Interquartile Range = 22) and medical attendants had the lowest scores (Median = 27.78, Interquartile Range = 39) as shown in S2 Fig. However, the difference in knowledge score shown in the boxplots was not statistically significant (Kruskal-Wallis test = 5.076, p-value = 0.166).

#### Domain 6: Knowledge on management of dengue and surveillance procedure

Knowledge about the management of dengue and surveillance procedures was lacking among the respondents. Only half of the participants (148, 50.7%) correctly identified paracetamol as the drug for managing fever and pain in dengue patients, while the rest chose incorrect responses such as ibuprofen (19, 9.2%), aspirin (19, 9.2%), all of the above (107,36.6%), or the answer “I don’t know” (10, 3.4%) as indicated in S4 Table. Less than half of the respondents (124, 42.5%) knew that there was no specific drug for treating dengue. Most respondents (237, 81.2%) were aware that intravenous fluid therapy is used to manage dengue symptoms. Of those who agreed to the use of intravenous fluid, only about half correctly identified the circumstances for prescribing it, such as for patients with severe dengue (123, 51.9%) and for patients who cannot tolerate oral fluid intake (108, 45.6%).

Only slightly over half of the respondents (159, 54.5%) were aware that blood transfusion can be given to dengue patients, and out of those, only 76(47.8%) knew that it is given during severe bleeding, as shown in S4 Table. Respondents showed better knowledge when asked about which dengue patients require hospitalization, with correct answers being chosen by 162(55.5%) for patients with severe dengue, 152(52.1%) for patients with warning signs, and 32(11.0%) for patients with special conditions such as pregnant women and children. See S4 Table for details on other responses provided by the respondents.

Based on boxplots analysis, clinicians had the highest knowledge score (Median = 46.15, Interquartile Range = 23). Laboratory personnel and nurses had similar median scores, but Interquartile ranges indicated better performance for nurses (Median = 38.46, Interquartile Range = 23) compared to laboratory personnel (Median = 38.46, Interquartile Range = 31). Medical attendants had the lowest score (Median = 30.77, Interquartile Range = 35) as shown in S2 Fig. However, the difference in knowledge score shown in the boxplots was not statistically significant (Kruskal-Wallis test = 10.492, p-value = 0.015).

### Domains comparison

**Fig 2:**
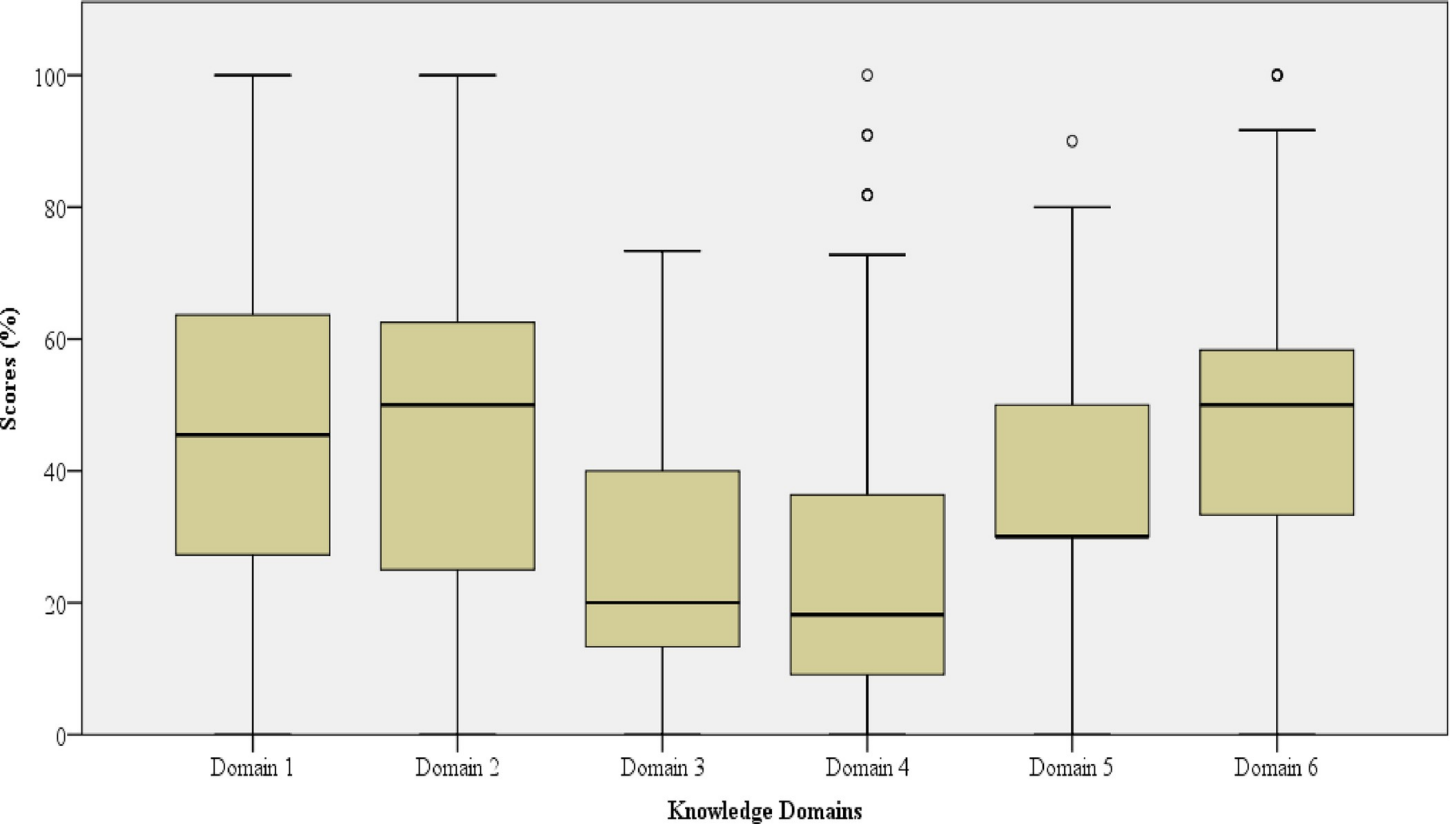
Boxplots-showing knowledge scores across all six domains. Domain 1: symptoms of dengue fever, domain 2: cause, transmission and prevention of dengue, domain 3: warning signs and risk factors for severe dengue, domain 4: dengue shock syndrome and associated symptoms, domain 5: diagnostic of dengue, domain 6: management of dengue and surveillance procedure.

As shown in Fig 2, boxplots demonstrated a higher median score for domain 6 (Median = 50.00, Interquartile Range = 25) followed by domain 2 (Median = 50.00, Interquartile Range = 38) and subsequently domain 1 (Median = 45.45, Interquartile Range = 36). On the other hand, domain 5, 3 and 4 had the lowest scores respectively (Median = 30.00, Interquartile Range = 20), (Median = 20.00, Interquartile Range = 27) and (Median = 18.18, Interquartile Range = 27).

### Overall knowledge score on dengue by health care workers

**Fig 3:**
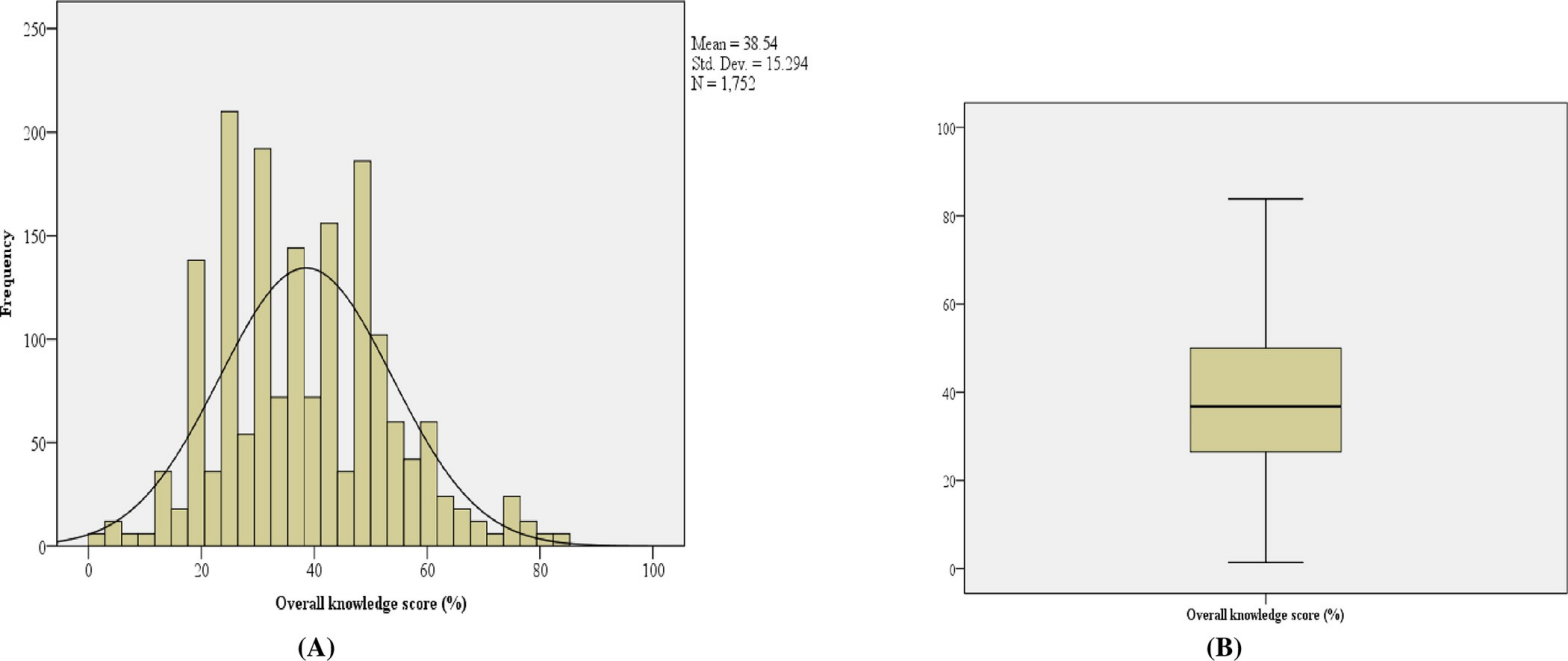
(A) Histogram showing overall knowledge score of dengue, (B) Boxplot showing Overall knowledge score of dengue.

Cumulative knowledge scores were computed for each respondent. The highest knowledge score was 84 and the lowest was 1. As shown in Fig 3, the mean score was 38.54 (standard deviation of 15.3), the median score was 36.76 (Interquartile Range of 24). The data were not normally distributed (Shapiro-wilk test, 0.984, p-value 0.002).

### Influence of socio-demographic factors on knowledge level

After categorizing the knowledge score of participants into binary variables, poor versus good, more than half the respondents had poor knowledge score on dengue (155, 53.1%). Based on Pearson Chi-Square test, variables such as, gender, qualification of participants, education and type of facility were associated with the level of knowledge of health care workers on dengue (p-value <0.005). The magnitude of association was further quantified by logistic regression (Table 4). Variables such as gender, qualification of participants, education, and type of health facility were each associated with a good knowledge level, p-value≤ 0.05 (Table 4).

**Table 4:** Predictors of good knowledge among health workers in Dar es Salaam region (N = 292).

	Univariate analysis		Multivariate analysis	
Variable	COR[95%CI]	P-value	AOR[95%CI]	P-value
**Gender**				
Male	1.662[1.044–2.644]	0.032*	1.043[0.615–1.768]	0.876
Female	0.699(ref)			
**Age**				
<40 years	0.908[0.486–1.696]	0.762	-	-
≥40 years	0.958(ref)			
**Qualification**				
Clinician	3.883[2.351–6.414]	0.000*	3.637[2.019–6.550]	0.000*
Laboratory/nurse/attendant	0.529(ref)			
**Education**				
Diploma/degree	2.405[1.441–4.014]	0.001*	1.324[0.714–2.454]	0.374
Certificate/short course	0.484(ref)			
**Experience in the profession**				
≤5 years	1.124[0.692–1.827]	0.636	-	-
≥6 years	0.818(ref)			
**Work station**				
Dispensary	0.664[0.398–1.107]	0.116	-	-
Health centre/hopsital/polyclinic	1.184(ref)			
**Type of facility**				
Private	1.783[1.098–2.894]	0.019*	2.071[1.220–3.514]	0.007*
Government	1.091(ref)			

*Statistically significant (p-value≤0.05), COR = Crude odds ratio, AOR = Adjusted odds ratio

Multivariate logistic regression identified qualification and type of health facility as significantly associated with good knowledge of health care workers on dengue. Specifically, as shown in Table 4, clinicians were almost four times more likely to have good knowledge compared to laboratory personnel /nurse/medical attendants (AOR, 3.637; CI, 2.019–6.550; p-value ≤ 0.0001). Health workers from private health facilities were two times more likely to have good knowledge compared to those from public health facilities (AOR, 2.071; CI, 1.220–3.514; p-value = 0.007).

### Health facilities preparedness in detection of dengue outbreaks

All 292 respondents were asked about the availability of rapid test/machines/guidelines necessary for dengue. Nearly a quarter of the respondents reported that dengue rapid tests were either only available during an outbreak period (107, 36.6%) or not available at all (113, 38.7%). Only 172(58.9%) respondents reported the availability of full blood count machines as shown in Table 5.

**Table 5:** Health facilities’ preparedness for detection of dengue outbreaks (N = 292).

Variable	Response	Government	Private	Frequency (%)	Chi-square value	P-value
**Dengue rapid tests**	Available all times	5(20.0%)	20(80.0%)	25(8.6)	0.328	0.955
	Available during an outbreak	43(40.2)	64(59.8)	107(36.6)		
	Not available at all	36(31.9)	77(68.1)	113(38.7)		
	I don’t know	24(51.1)	23(48.9)	47(16.1)		
**Full blood count machine**	Available	83(48.3)	89(51.7)	172(58.9)	1.640	0.650
	Not available	20(18.5)	88(81.5)	108(37.0)		
	Available but not functional	1(25.0)	3(75.0)	4(1.4)		
	I don’t know	4(50.0)	4(50.0)	8(2.7)		
**Dengue guidelines**	Available all times	48(46.2)	56(53.8)	104(35.6)	2.482	0.478
	Available during an outbreak	10(28.6)	25(71.4)	35(12.0)		
	Not available at all	28(25.7)	81(74.3)	109(37.3)		
	I don’t know	22(50.0)	22(50.0)	44(15.1)		
**Refresher training on dengue**	Available all times	21(48.8)	22(51.2)	43(14.7)	1.426	0.700
	Available during an outbreak	14(35.9)	25(64.1)	39(13.4)		
	Not available at all	63(33.2)	127(66.8)	190(65.1)		
	I don’t know	10(50.0)	10(50.0)	20(6.8)		
**Standard case definitions for dengue**	Available all times	21(48.8)	22(51.2)	43(14.7)	5.937	0.115
	Available during an outbreak	45(36.3)	79(63.7)	124(42.5)		
	Not available at all	20(27.0)	54(73.0)	74(25.3)		
	I don’t know	22(43.1)	29(56.9)	51(17.5)		

Only a minority of respondents reported that dengue guidelines were available at all times (104, 35.6%), while a slightly higher number reported that they were not available at all (109, 37.3%). Most of the respondents (190, 65.1%) reported that refresher training on dengue was not available at all times, and only 124 (42.5%) reported that case definitions for dengue were available during an outbreak. A considerable proportion of respondents answered "I don’t know" for the availability of dengue rapid tests (47, 16.1%), full blood count machine (8, 2.7%), dengue guidelines (44, 15.1%), refresher training (20, 6.8%), and standard case definitions for dengue (51, 17.5%), as presented in Table 5. Other responses chosen by participants are as shown in Table 5.

As shown in Table 5, there was no statistically significance difference regarding availability of dengue rapid tests, full blood count machines, dengue guidelines, refresher trainings on dengue as well as standard case definitions.

## Discussion

The study analyzed the level of knowledge of health care workers about dengue infection and health facilities preparedness for the detection of dengue in the Dar es Salaam region, Tanzania. The findings of this study revealed the following major gaps that warrant intervention measures.

First, our findings show a lack of adequate knowledge among healthcare workers, specifically concerning severe dengue. This was demonstrated by low median scores on the topic of warning signs (Median = 20.00, Interquartile Range = 27) and risk factors for severe dengue as well as symptoms of dengue shock syndrome (Median = 18.18, and Interquartile Range = 27). Similar to our study, in Bhutan, Asia, few health care workers were knowledgeable of severe dengue outcomes [44]. In previous outbreaks, dengue patients rarely progressed to severe disease outcomes, possibly causing a lack of awareness about this form of disease. However, the 2019 outbreak saw an increase in severe dengue cases [11], emphasizing the need to train health care workers on severe dengue illness.

Second, our study reports a gap on knowledge level of dengue between clinicians and support staff. Clinicians were almost four times more likely to have good knowledge (score ≥ 40%) than laboratory personnel /nurse/medical attendants (AOR, 3.637; p-value 0.000). These findings agree with previous surveys in Ethiopia, Bhutan, Togo, United States, Vietnam as well as Pakistan and Egypt [37, 44, 45, 48, 49, 65]. While this outcome seems expected, the importance of support staff should not be understated. Nurses and laboratory personnel are involved with patient care and have equal contribution to the patient outcomes. While clinicians are responsible for planning diagnoses and treatments, it falls to laboratory personnel to conduct the confirmatory tests. Nurses take on the role of implementing treatments, monitoring vital signs and overseeing progress of patients. In regions facing a shortage of healthcare workers, roles typically carried out by clinicians are often taken up by nurses, laboratory personnel, and sometimes medical attendants [30, 31, 66]. In general, good patient outcomes are a result of interdisciplinary collaboration between clinicians and support staffs [67–70]. Consequently, it is necessary that all health care workers involved in patient care have comprehensive knowledge of dengue to contribute effectively.

What is more, our results show the lack of resources necessary for diagnosis of dengue, especially during non-outbreak periods. Beyond outbreak periods, dengue rapid tests and dengue guidelines were reported to be accessible to only 8.6% and 35.6% of the respondents, respectively. Previous studies also reported inadequate availability of diagnostic tests [22, 23, 45]. It is certain that absence of confirmatory tests in health facilities is due to unaffordable costs [71, 72]. Clinical presentation of dengue is similar to other febrile illnesses and diagnosis without confirmatory test is difficult [9]. However, without correct diagnostics, any outbreak is sure to be detected late if at all posing a considerable public health risk in a resource limited country. To start the use of transmission control measures, an outbreak has to be reported first and therefore diagnostic tests, guidelines and standard case definitions should be accessible at all times. They promote standardized diagnosis, allow timely treatment and control of spread of infectious diseases, provide accurate measures to morbidity and mortality rates, allow standardization of service offered within epidemic and inter epidemic periods and provide quality data in the diseases notification systems [73, 74].

Lastly, our study found that refresher training for dengue was inadequate during non-outbreaks (14.7%). These findings partly explain our results on inadequate knowledge of dengue discussed in the previous sections. This was also discovered in previous studies that reported limited trainings on dengue among health care workers in Togo and Northern Tanzania [23, 45]. It must be emphasized that refresher training plays a crucial role in outbreak preparedness by helping healthcare workers learn or regain knowledge and skills that they may have forgotten over time [13, 75].

## Conclusion

For a country facing regular and increasingly severe dengue outbreaks, Tanzania does not seem to be well prepared. The study revealed inadequate knowledge about dengue among health care workers primarily concerning the recognition of warning signs for severe dengue and dengue shock syndrome. Disparities in dengue knowledge were evident, with clinicians demonstrating better knowledge compared to other cadres of health care workers. Beyond health care workers’ knowledge on dengue, the study revealed a lack of readiness in terms of diagnostic tools and training across both privately owned and government-run health facilities. Considering dengue’s endemicity in Tanzania and the potential impact of climate change on its incidence, we recommend incorporating lessons on emerging infectious diseases into health care training curricula, providing regular in-service training, establishing a practice of continuous learning through recognized online platforms, and assisting developing nations like Tanzania in accessing crucial diagnostic equipment. A comprehensive countrywide knowledge study using mixed-methods is recommended, along with future research to investigate the efficacy of educational interventions and the development of simplified dengue guidelines.

## Limitation of the study

The results of this study may not be generalizable to all health care workers in the Dar es Salaam region because it was limited to few health facilities in one district out of the five districts in the region. Moreover, it relied on convenience sampling rather than random probability sampling techniques. Furthermore, there were 10% non-response rates, which may have caused response bias. Despite these limitations, this study is the first to evaluate the knowledge of health care workers regarding dengue and the preparedness of health facilities for dengue detection in Dar es Salaam city, which experiences regular outbreaks. We anticipate that this study will serve as the baseline for subsequent countrywide knowledge surveys. The knowledge gaps will be addressed in order to improve diagnostics and surveillance actions for dengue and other emerging and reemerging infections in the country.

## Supporting information

S1 Fig Sampling procedure. (DOCX)

S2 Fig Boxplots showing difference in knowledge score among health care workers across different knowledge domains. (DOCX)

S1 Table Knowledge of symptoms of dengue fever. (DOCX)

S2 Table Knowledge on causes, transmission and prevention of dengue. (DOCX)

S3 Table Knowledge on diagnosis of dengue. (DOCX)

S4 Table Knowledge on management of dengue symptoms and surveillance procedure. (DOCX)

S5 Table Variation in health workers’ level of knowledge towards dengue among different groups of respondents based on Pearson Chi-Square test. (DOCX)
