# Supplementary material for: Health professionals’ knowledge on dengue and health facility preparedness for case detection: A cross-sectional study in Dar es Salaam, Tanzania
Source: PLoS Negl Trop Dis. 2023 Nov 21;17(11):e0011761. doi: 10.1371/journal.pntd.0011761 (PMC10662763; doi:10.1371/journal.pntd.0011761)
Supplement: S1 Fig — (DOCX) [file pntd.0011761.s001.docx]

Excluded (N=32)

Reason: 18 incomplete questionnaires, 14 never returned questionnaires and all claimed to be due to shortage of time

Health facilities available in Temeke district as per district medical office statistics of 2021 (N=166)

Public =24

Private= 142

Health facilities meeting inclusion criteria (N=148)

Public=22

Private=127

Inclusion criteria: offering generalized clinical services (outpatient and/or inpatient)

Convenience sampling (access to location details and consent from health facility administrators for staff participation in the study) was used to select health facilities to be included in the study (N=78)

Public=22

Private=56

Excluded (N=17)

Reason: offering specialized clinics only, examples, eye clinic, dental clinic etc and those offering diagnostic facilities/laboratory services only

Taking part in pilot phase

Health care workers included in analysis (N= 292)

Convenience sampling (availability at the time of health facility visits) was used to select health care workers to be included in the study (N=324)

Public=108

Private=184

Inclusion criteria: age ≥18 years, readiness to sign consent forms, filling questionnaire within 24 hours

Temeke district was selected by simple random sampling

Dar es Salaam Region (5 districts: Ilala, Kigamboni, Kinondoni, Temeke and Ubungo)

**Fig S1: Sampling procedure**
