# Supplementary material for: Health professionals’ knowledge on dengue and health facility preparedness for case detection: A cross-sectional study in Dar es Salaam, Tanzania
Source: PLoS Negl Trop Dis. 2023 Nov 21;17(11):e0011761. doi: 10.1371/journal.pntd.0011761 (PMC10662763; doi:10.1371/journal.pntd.0011761)
Supplement: S2 Fig — (DOCX) [file pntd.0011761.s002.docx]

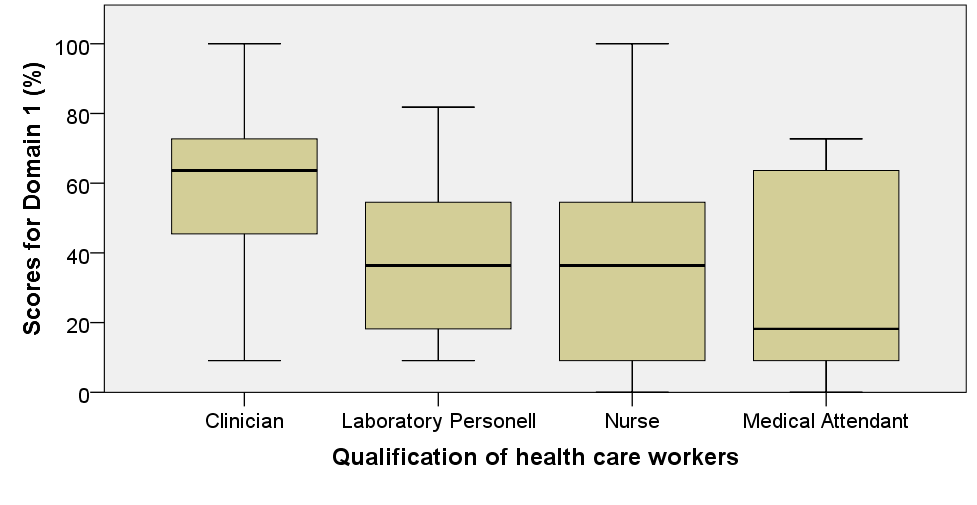

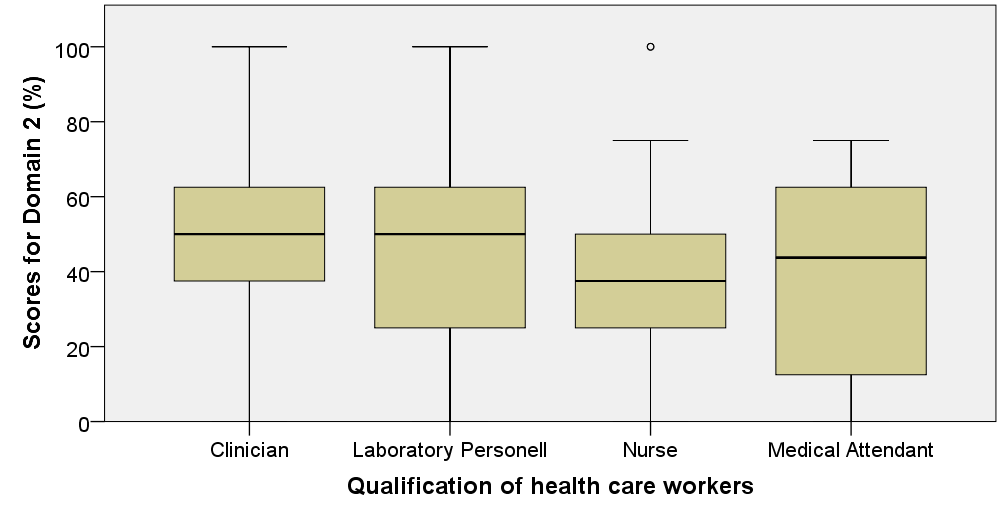
 **(A) (B)**

**
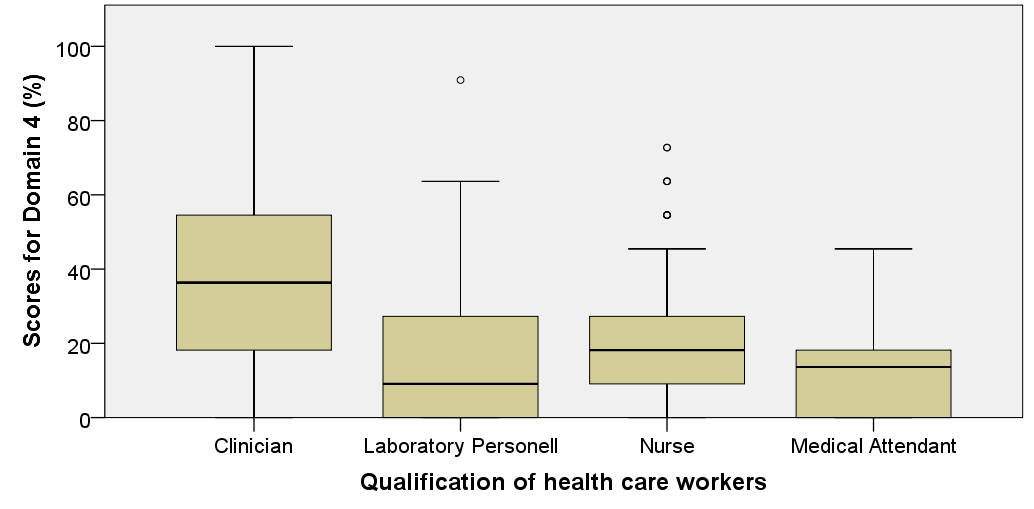
**
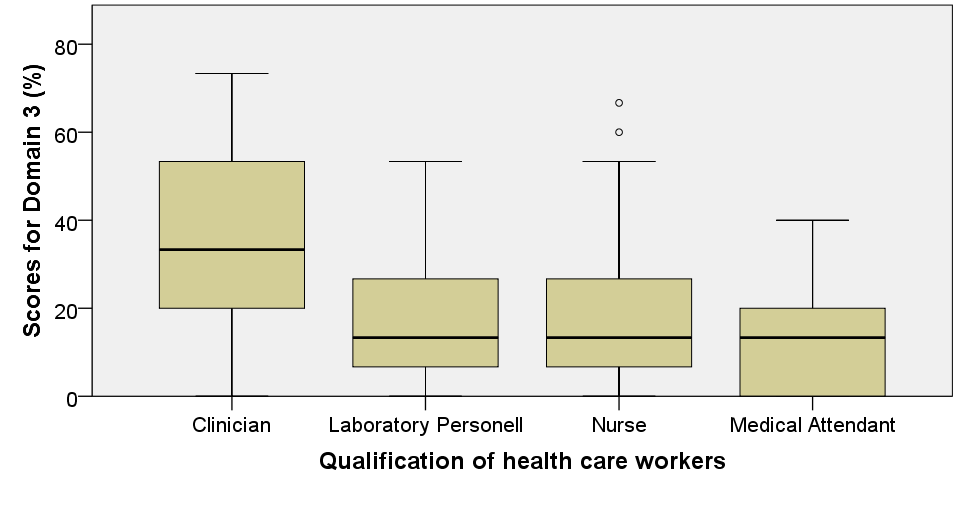


**
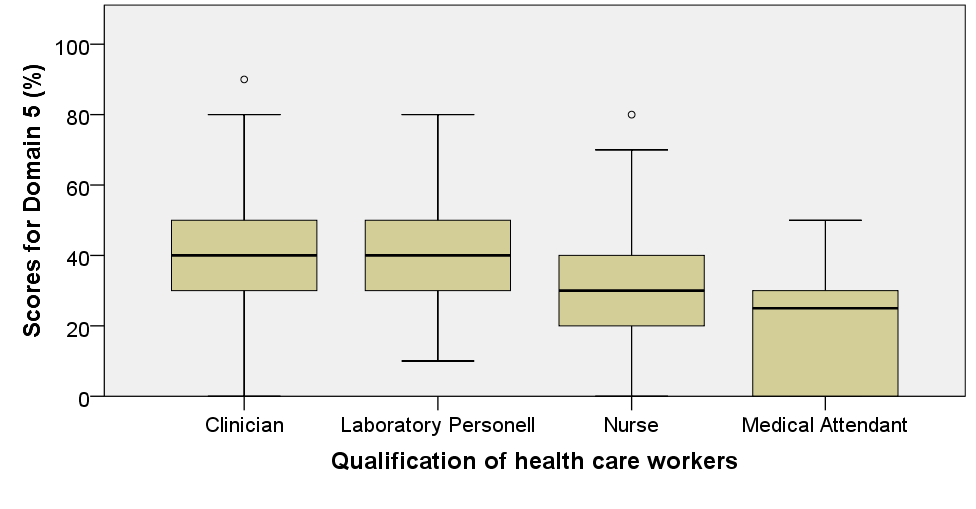

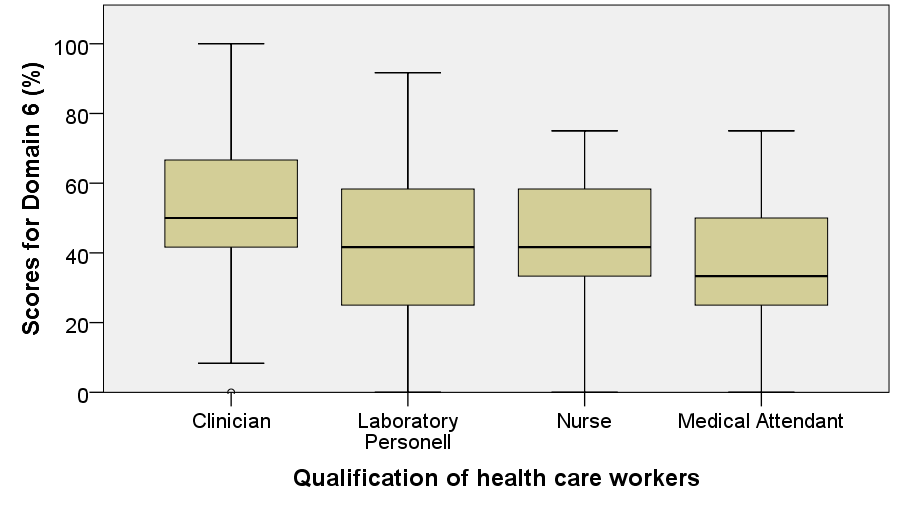
 (C) (D)**

**(E) (F)**

**Fig S2. Boxplots showing difference in knowledge score among health care workers across different knowledge domains.** Box plot (1) Clinicians, (2) Laboratory personnel, (3) Nurses, (4) Medical Attendants. (A) Domain 1: symptoms of dengue fever, (B) Domain 2: cause, transmission and prevention of dengue (C) Domain 3: warning signs and risk factors for severe dengue (D) Domain 4: dengue shock syndrome and associated symptoms (E) Domain 5: diagnostic of dengue (F) Domain 6: management of dengue and surveillance procedure
