# Supplementary material for: Health professionals’ knowledge on dengue and health facility preparedness for case detection: A cross-sectional study in Dar es Salaam, Tanzania
Source: PLoS Negl Trop Dis. 2023 Nov 21;17(11):e0011761. doi: 10.1371/journal.pntd.0011761 (PMC10662763; doi:10.1371/journal.pntd.0011761)
Supplement: S1 Table — (DOCX) [file pntd.0011761.s003.docx]

**S1 Table. Health workers awareness on dengue and common dengue symptoms (N=292).**

| **Variable** | **Proportion of respondent answering “Yes” (%)** |
| --- | --- |
| Heard about dengue | 291(99.7) |
| **Symptoms of dengue fever** | |
| High fever | 267(91.4) |
| Headache | 223(76.4) |
| Loss of appetite | 153(52.4) |
| Vomiting | 142(48.6) |
| Muscle pain | 168(57.5) |
| Joint pain | 174(59.6) |
| Pain behind eyes | 81(27.7) |
| Rashes | 69(23.6) |
| Petechiae | 55(18.8) |
| Gums and nose bleed | 53(18.2) |
| Bruising at venipuncture site | 44(15.1) |
| I don’t know any symptom | 3(1.0) |

**All symptoms listed here are probable symptoms of dengue fever**
