# Supplementary material for: Health professionals’ knowledge on dengue and health facility preparedness for case detection: A cross-sectional study in Dar es Salaam, Tanzania
Source: PLoS Negl Trop Dis. 2023 Nov 21;17(11):e0011761. doi: 10.1371/journal.pntd.0011761 (PMC10662763; doi:10.1371/journal.pntd.0011761)
Supplement: S2 Table — (DOCX) [file pntd.0011761.s004.docx]

**S2 Table. Knowledge on causes, transmission and prevention of dengue (N=292)**

| **Variable** | **Responses** | **Frequency (%)** |
| --- | --- | --- |
| **Pathogen that causes dengue** | Bacteria | 7 (2.4) |
|  | Parasite | 121 (41.4) |
|  | Virus^+^ | 128 (43.8) |
|  | Protozoa | 16 (5.5) |
|  | I don’t know | 20 (6.8) |
|  | Total | 292 (100)^*^ |
| **Methods that transmit dengue** | | |
| Sexual intercourse | Yes | 3 (1.0) |
| Mosquito bite^₊^ | Yes | 259 (88.7) |
| Tsetse fly bite | Yes | 35 (12.0) |
| Contaminated foods and drinks | Yes | 7 (2.4) |
| Contact with infected human or animals | Yes | 18 (6.2) |
| Blood transfusion^₊^ | Yes | 46 (15.8) |
| Maternal transmission^₊^ | Yes | 30 (10.3) |
| I don’t know any method | Yes | 4 (1.4) |
| **Methods to avoid dengue** | | |
| Sleeping under mosquito nets^₊^ | Yes | 219 (75.0) |
| Covering water containers^₊^ | Yes | 126 (43.2) |
| Avoid mosquito bites^₊^ | Yes | 175 (59.9) |
| Empty water holding containers around the house^₊^ | Yes | 113 (38.7) |
| I don’t know any method | Yes | 7 (2.4) |

**^+^ Symbol represents the correct answers**

**^*^ Symbol means decimals have been rounded**
