# Supplementary material for: Health professionals’ knowledge on dengue and health facility preparedness for case detection: A cross-sectional study in Dar es Salaam, Tanzania
Source: PLoS Negl Trop Dis. 2023 Nov 21;17(11):e0011761. doi: 10.1371/journal.pntd.0011761 (PMC10662763; doi:10.1371/journal.pntd.0011761)
Supplement: S3 Table — (DOCX) [file pntd.0011761.s005.docx]

**S3 Table: Knowledge on Diagnosis of Dengue (N=292)**

| **Variable** | **Proportion of respondent answering “Yes” (%)** |
| --- | --- |
| **Tests used to confirm dengue** | |
| Rapid test | 218(74.7) |
| Enzymes Linked Immunosorbent Assay (ELISA) | 46(15.8) |
| Polymerase Chain Reaction (PCR) | 75(25.7) |
| Blood culture | 50(17.1) |
| **Samples used to diagnose dengue** | |
| Whole blood | 216(74.0) |
| Serum | 125(42.8) |
| Plasma | 125(42.8) |
| **Other tests used to identify probable dengue cases** | |
| Tourniquet test | 14(4.8) |
| Full blood count | 198(67.8) |

**All tests and samples listed in Table 6 are correct responses**
