# Supplementary material for: Health professionals’ knowledge on dengue and health facility preparedness for case detection: A cross-sectional study in Dar es Salaam, Tanzania
Source: PLoS Negl Trop Dis. 2023 Nov 21;17(11):e0011761. doi: 10.1371/journal.pntd.0011761 (PMC10662763; doi:10.1371/journal.pntd.0011761)
Supplement: S4 Table — (DOCX) [file pntd.0011761.s006.docx]

**S4 Table. Knowledge on management of dengue symptoms and surveillance procedure**

| **Variable** | **Responses** | **Frequency (%)** |
| --- | --- | --- |
| Drug used for the management of fever and pain in dengue patients (N=292) | Paracetamol^₊^ | 148(50.7) |
|  | Ibuprofen | 19(9.2) |
|  | Aspirin | 19(9.2) |
|  | All of the above | 107(36.6) |
|  | I don’t know | 10(3.4) |
| Specific drug used to treat dengue infection (N=292) | Antiparasitic drugs | 57(19.5) |
|  | Antiviral drugs | 27(9.2) |
|  | Antibiotic drugs | 16(5.7) |
|  | dengue vaccine | 12(4.1) |
|  | No specific drug^₊^ | 124(42.5) |
|  | I don’t know | 56(19.2) |
| Intravenous fluid therapy is used to treat dengue symptoms (N=292) | Yes₊ | 237(81.2) |
|  | No | 13(4.5) |
|  | I don’t know | 42(14.4) |
| Dengue patients that need intravenous fluids (N=237) |  |  |
| All confirmed dengue cases | Yes | 70(29.5) |
|  | No | 167(70.5) |
| All suspect dengue cases | Yes | 19(8.0) |
|  | No | 218(92.0) |
| Probable dengue cases | Yes | 14(5.9) |
|  | No | 223(94.1) |
| Patients with severe dengue^₊^ | Yes | 123(51.9) |
|  | No | 114(48.1) |
| Patients that cannot tolerate oral fluid intake^₊^ | Yes | 108(45.6) |
|  | No | 129(54.4) |
| Blood transfusion therapy is used to treat dengue symptoms (N=292) | Yes₊ | 159(54.5) |
|  | No | 76(26.0) |
|  | I don’t know | 57(19.5) |
| Dengue patients that need blood transfusion (N=159) |  |  |
| All patients with bleeding symptoms | Yes | 40(25.2) |
|  | No | 119(74.8) |
| All patients with low platelet count | Yes | 82(51.6) |
|  | No | 77(48.4) |
| All patients with severe bleeding^₊^ | Yes | 76(47.8) |
|  | No | 86(52.2) |
| All dengue suspect cases | Yes | 7(4.4) |
|  | No | 152(95.6) |
| All patients in dengue shock | Yes | 10(6.3) |
|  | No | 149 (93.7) |
| Dengue patients admitted for hospitalization (N=292) |  |  |
| All suspect cases | Yes | 68(23.3) |
|  | No | 224(76.7) |
| Pregnant women and children^₊^ | Yes | 32(11.0) |
|  | No | 260(89.0) |
| Patients with warning signs^₊^ | Yes | 152(52.1) |
|  | No | 140(47.9) |
| Patients with severe disease^₊^ | Yes | 162(55.5) |
|  | No | 130(44.5) |
| I don’t know | Yes | 8(2.7) |
|  | No | 284(97.3) |
| Informed about the need to report dengue suspect cases (N=292) | Yes^+^ | 146(50.0) |
|  | No | 146(50.0) |
| Aware of a dengue case definition in Tanzania (N=292) | Yes^₊^ | 172(58.9) |
|  | No | 120(41.1) |

**^+^ Symbol represents the correct answers**
