# Supplementary material for: Health professionals’ knowledge on dengue and health facility preparedness for case detection: A cross-sectional study in Dar es Salaam, Tanzania
Source: PLoS Negl Trop Dis. 2023 Nov 21;17(11):e0011761. doi: 10.1371/journal.pntd.0011761 (PMC10662763; doi:10.1371/journal.pntd.0011761)
Supplement: S5 Table — (DOCX) [file pntd.0011761.s007.docx]

**S5 Table. Variation in health workers’ level of knowledge towards dengue among different groups of respondents based on Pearson Chi-Square test (N=292)**

| **Variable** | **Category** | **Poor knowledge** | **Good knowledge** | **X^2^ (df)** | **P-value** |
| --- | --- | --- | --- | --- | --- |
| **Gender** | Male | 62(46.3) | 72(53.7) | 4.616(1) | 0.032^*^ |
|  | Female | 93(58.9) | 65(41.1) |  |  |
| **Age** | <40 years | 131(53.5) | 114(46.5) | 0.092(1) | 0.762 |
|  | ≥40 years | 24(51.1) | 23(48.9) |  |  |
| **Qualification** | Clinician | 36(32.7) | 74(67.3) | 29.360 (1) | 0.000^*^ |
|  | Laboratory/nurse/attendant | 119(65.4) | 63(34.6) |  |  |
| **Education** | Diploma/degree | 91(46.2) | 106(53.8) | 11.541 (1) | 0.001^*^ |
|  | Certificate/short course | 64(67.4) | 31(32.6) |  |  |
| **Experience in the profession** | ≤5 years | 100(52.1) | 92(47.9) | 0.225 (1) | 0.636 |
|  | ≥6 years | 55(55.0) | 45(45.0) |  |  |
| **Work station** | Dispensary | 117(56.0) | 92(44.0) | 2.481(1) | 0.115 |
|  | Health centre/hopsital/polyclinic | 38(45.8) | 45(54.2) |  |  |
| **Type of facility** | Government | 67(62.0) | 41(38.0) | 5.518(1) | 0.019^*^ |
|  | Private | 88(47.8) | 96(52.2) |  |  |

**^*^Statistically significant (p < 0.05), X^2^ stands for chi-square value, (df) stands for degrees of freedom**
